# Characterization of a novel aspartic protease from *Trichoderma asperellum* for the preparation of duck blood peptides

**DOI:** 10.1007/s00253-023-12848-y

**Published:** 2024-01-13

**Authors:** Yibin Xue, Qiaojuan Yan, Xue Li, Zhengqiang Jiang

**Affiliations:** 1 Key Laboratory of Food Bioengineering (China National Light Industry), College of Food Science & Nutritional Engineering, China Agricultural University, Beijing, 100083 China; 2 College of Engineering, China Agricultural University, Beijing, 100083 China; 3 Food Laboratory of Zhongyuan, Luohe City, 462000 Henan Province China

**Keywords:** *Trichoderma asperellum*, Aspartic protease, *Komagataella phaffii*, Biochemical characterization, Duck blood peptides

## Abstract

**Abstract:**

A novel aspartic protease gene (*Tapro*A1) from *Trichoderma asperellum* was successfully expressed in *Komagataella phaffii* (*Pichia pastoris*). *Ta*proA1 showed 52.8% amino acid sequence identity with the aspartic protease PEP3 from *Coccidioides posadasii* C735. *Ta*proA1 was efficiently produced in a 5 L fermenter with a protease activity of 4092 U/mL. It exhibited optimal reaction conditions at pH 3.0 and 50 °C and was stable within pH 3.0–6.0 and at temperatures up to 45 °C. The protease exhibited broad substrate specificity with high hydrolysis activity towards myoglobin and hemoglobin. Furthermore, duck blood proteins (hemoglobin and plasma protein) were hydrolyzed by *Ta*proA1 to prepare bioactive peptides with high ACE inhibitory activity. The IC_50_ values of hemoglobin and plasma protein hydrolysates from duck blood proteins were 0.105 mg/mL and 0.091 mg/mL, respectively. Thus, the high yield and excellent biochemical characterization of *Ta*proA1 presented here make it a potential candidate for the preparation of duck blood peptides.

**Key points:**

• *An aspartic protease (TaproA1) from Trichoderma asperellum was expressed in Komagataella phaffii.*

• *TaproA1 exhibited broad substrate specificity and the highest activity towards myoglobin and hemoglobin.*

• *TaproA1 has great potential for the preparation of bioactive peptides from duck blood proteins.*

**Supplementary Information:**

The online version contains supplementary material available at 10.1007/s00253-023-12848-y.

## Introduction

Proteins are hydrolyzed by proteases (EC 3.4.11-24) to yield amino acids and bioactive peptides. Aspartic proteases (EC 3.4.23), commonly called acid proteases, have been widely used in different foods, such as cheese, bread, beverages, meat, and bioactive peptides (Mamo and Assefa 2018; Rocha et al. 2021).

They have two highly conserved aspartic acid residues located at the center of the active site responsible for their catalytic activity (Guo et al. 2020). Pepsin-like and chymosin-like aspartic proteases are the two main types of aspartic proteases. Pepsin-like aspartic proteases are mainly derived from *Trichoderma* sp., *Penicillium* sp., and *Aspergillus* sp., while chymosin-like aspartic proteases are mainly derived from *Endothia* sp., *Rhizopus* sp., and *Mucor* sp. (Da Silva et al. 2016; Siala et al. 2009). Generally, aspartic proteases have optimal pH values within the range of pH 2.0–6.0 and are stable in an acid environment (Horimoto et al. 2009). They prefer to cleave peptide bonds between residues with hydrophobic side chains, such as Leu-Tyr, Phe-Phe, and Phe-Tyr (Mandujano-González et al. 2016). Most reported aspartic proteases are mesophilic and show optimal temperatures between 40 ℃ and 60 °C (Souza et al. 2017; Yang et al. 2013). Aspartic proteases are first synthesized as inactive precursors (zymogens), which can effectively protect the proteases from damage caused by the body (Dunn 2002). They are usually autocatalytically activated under acid conditions (Guo et al. 2020). Besides, aspartic proteases have broad substrate specificity, and their activities are inhibited by pepstatin A (Guo et al. 2021; Souza et al. 2017).

Currently, cloning and expression of protease genes by genetic engineering technology are effective ways to identify novel proteases. To date, various proteases have been successfully expressed in *Komagataella phaffii* (*Pichia pastoris*) (Guo et al. 2021; Mechri et al. 2021; Song et al. 2021). Microorganisms are the preferred sources of proteases, owing to their rapid growth, simple cultivation, and convenience for genetic manipulation (Mamo and Assefa 2018). The alkaline serine protease from *Trichoderma koningii* expressed in *K. phaffii* displayed enzyme activity of 15,900 U/mL (Shu et al. 2016). The neutral protease from *Aspergillus oryzae* expressed in *K. phaffii* exhibited enzyme activity of 43,101 U/mL (Ke et al. 2012). However, the expression level of acid proteases (aspartic proteases) in *K. phaffii* is relatively low. The aspartic protease from *A. repens* expressed in *K. phaffii* showed enzyme activity of only 1.4 U/mL (Takenaka et al. 2017). Two aspartic proteases from *Talaromyces leycettanus* and *Penicillium* sp. XT7 expressed in *K. phaffii* displayed enzyme activities of 67.8 U/mL and 89.3 U/mL, respectively (Guo et al. 2019; Guo et al. 2021). The aspartic protease from *Trichoderma harzianum* expressed in *K. phaffii* exhibited enzyme activity up to 328.1 U/mL (Deng et al. 2018). The aspartic protease from *A. niger* expressed in *K. phaffii* showed enzyme activity of 1500 U/mL (Wei et al. 2023). Thus, high-level expression of aspartic proteases has great application potential.

Bioactive peptides are functional short-chain amino acid sequences that alleviate diseases and have no side effects on human health (Singh et al. 2022). Compared with antihypertensive drugs, angiotensin-I-converting enzyme (ACE) inhibitory peptides produced by the enzymatic hydrolysis of food-derived proteins have become a preferred choice to lower blood pressure due to their safety and no side effects (Gomes et al. 2020). Duck blood has been applied to improve economic values in the preparation of bioactive peptides, such as antioxidant peptides (Yang et al. 2020) and ACE inhibitory peptides (Wang et al. 2021). However, most of the proteases used for the preparation of ACE inhibitory peptides are commercial proteases such as pepsin, trypsin, papain, and bromelain. Therefore, the exploration of novel proteases for the preparation of bioactive peptides will attract much attention.

In this study, a novel aspartic protease gene (*Tapro*A1) from *Trichoderma asperellum* was cloned and expressed in *K. phaffii*. Fed-batch fermentation was performed for the production of *Ta*proA1 in a 5 L fermenter. *Ta*proA1 was further purified and characterized. Moreover, its valuable application potential was evaluated for the preparation of duck blood peptides with high ACE inhibitory activity. This study aims to provide a suitable protease for the enzymatic conversion of duck blood proteins.

## Materials and methods

### Strains, plasmids, and reagents

*T. asperellum* CAU126 was screened, identified, and preserved in China General Microbiological Culture Collection Center (CGMCC No. 3.5921). *Escherichia coli* strain DH5α (TransGene, Beijing, China) was employed as the host for the cloning and sequencing of *Ta*proA1. The *K. phaffii* GS115 (his4, Mut^+^, Invitrogen) strain was the chassis host for *Ta*proA1 expression. pEASY-Blunt (TransGen, Beijing, China) and pPIC9K (Invitrogen, Carlsbad, CA, USA ) plasmids were utilized as the cloning and the expression vectors, respectively. FastPfu DNA polymerase, NEBbuilder® HiFi DNA Assembly Master Mix, and restriction enzymes (NEB, Frankfurt, Germany) were used for DNA manipulation. Duck blood hemoglobin and plasma protein were obtained from Handan Xinheng Biotechnology Co., Ltd. Casein sodium salt from bovine milk was purchased from Sigma-Aldrich (St. Louis, MO, USA), and all other reagents used herein were commercially accessible and analytical grade.

### Sequence analysis and expression of *Ta*proA1

The SignalP 4.1 server (http://www.cbs.dtu.dk/services/SignalP) predicted the *Ta*proA1 signal peptide sequence. The molecular weight and isoelectric point of the mature *Ta*proA1 protein were predicted using the ExPASy ProtParam tool (https://web.expasy.org/protparam/). Clustal Omega was applied to perform multiple sequence alignments (https://www.ebi.ac.uk/Tools/msa/clustalo/). NetNGlyc 1.0 (http://www.cbs.dtu.dk/services/NetNGlyc/) and NetOGlyc 4.0 (http://www.cbs.dtu.dk/services/NetOGlyc/) were used to analyze the glycosylation sites. The maximum likelihood method in MEGA 7.0 was used to construct the phylogenetic tree, which was then evaluated with 1000 bootstrap replicates (Kumar et al. 2016).

Genomic DNA and total RNA were extracted from the mycelia of *T. asperellum* using the fungal DNA and RNA Midi kit (TianGen, Beijing, China). The PrimeScript™ RT-PCR kit was used to reverse transcribe RNA into cDNA (Takara, Osaka, Japan). The *Tapro*A1 gene was amplified using DNA and cDNA as templates with the primers *Ta*proA1-F/R (Table S1). The PCR products were ligated into the pEASY-Blunt vector (TransGen, Beijing, China) for sequence analysis of the *Tapro*A1 gene.

The restriction enzymes *EcoR*I and *Not*I were utilized to digest the expression vector pPIC9K (Invitrogen, Carlsbad, CA, USA). The coding sequence (without the signal peptide) of *Tapro*A1 was fused into the digested pPIC9K plasmid to yield the recombinant vector pPIC9K-*Ta*proA1 by the seamless cloning method. Then, the plasmid pPIC9K-*Ta*proA1 was confirmed by DNA sequencing and linearized with the restriction endonuclease *Sac*I. The digested pPIC9K-*Ta*proA1 plasmid was electrically transformed into *K. phaffii* GS115 competent cells. The colonies were collected, and their genomes were extracted. The *Tapro*A1 gene was integrated into the *K. phaffii* GS115 genome using the two primers, 5′AOX1 and 3′AOX1 (Table S1)*.* These screened positive colonies were cultivated in the BMMY medium to express *Ta*proA1, and the protease activity was determined to verify the successful expression of *Ta*proA1 in the *K. phaffii* GS115 host.

### Production of *Ta*proA1 in a 5 L fermenter

According to the *K. phaffii* fermentation instruction manual (Invitrogen, San Diego, CA, USA), fed-batch fermentation was carried out by the engineering *K. phaffii* GS115 strain in a 5 L fermenter for the production of *Ta*proA1. The engineering *K. phaffii* GS115 strain was cultivated in the shake flask containing YPD medium until the OD_600_ was approximately 10.0 for inoculation. Batch culture, glycerol feeding culture, and 100% methanol induction culture were three stages that were performed during the whole fermentation process. At the methanol induction culture stage, the pH value was adjusted to pH 6.0 with 28% ammonia water, and 100% methanol was added to maintain the content of dissolved oxygen above 20%. The protease activity, protein content, and cell wet weight of the sample were determined during the fermentation phase.

### Purification of *Ta*proA1

The fermentation supernatant was obtained by centrifugation at 12,000 rpm and 4 °C for 10 min and then concentrated by the membrane package (10 kDa). The crude enzyme was dialyzed overnight in buffer A (20 mM phosphate buffer pH 6.0) and loaded on the Q-Sepharose Fast Flow (QSFF) column that was pre-equilibrated with buffer A. Unbound proteins were washed using buffer A, and a linear NaCl gradient in elution buffer B (20 mM phosphate buffer pH 6.0, 500 mM NaCl) was used to elute the bound *Ta*proA1 protein at a flow rate of 1.0 mL/min. Sodium dodecyl sulfate-polyacrylamide gel electrophoresis (SDS-PAGE, 12.5%) was used to analyze the purity of *Ta*proA1. The gel was stained with Coomassie brilliant blue R-250.

### Protease activity and protein content

*Ta*proA1 activity was determined according to the previously described method with minor modifications (Ichishima 1970). In brief, 100 μL of appropriately diluted *Ta*proA1 (50 mM citrate buffer pH 3.0) was mixed with 100 μL of casein (1%, w/v) solution (prepared in the same buffer) and incubated at 50 °C for 10 min. Then, the reaction was stopped by the addition of 200 μL of 0.4 M trichloroacetic acid (TCA) solution. After 3 min of centrifugation at 10,000 rpm, 100 μL of supernatant was mixed with 500 μL of 0.4 M sodium carbonate solution, followed by the addition of 100 μL of the Folin phenol reagent. After 20 min of incubation at 50 °C, the mixture was cooled to room temperature, and the absorbance was determined at 680 nm. One enzyme activity unit (U) was defined as the amount of protease required to hydrolyze casein to produce 1 μg tyrosine per min under the assay conditions.

The Lowry method was performed to determine the protein content, and bovine serum albumin (BSA) was used as the standard protein (Lowry 1951). The enzyme activity per milligram of protein was defined as the specific activity (U/mg).

### Biochemical characterization of *Ta*proA1

The optimal pH of *Ta*proA1 was determined by evaluating the protease activity in 50 mM buffers of various pH values (glycine-HCl, pH 1.5–3.0; citrate, pH 2.5–7.5; and Tris-HCl, pH 7.0–8.0 buffers). *Ta*proA1 was pre-incubated in the above buffers at 40 °C for 30 min to measure the pH stability. The optimal temperature of *Ta*proA1 was determined at different temperatures (30–70 °C) in 50 mM citrate buffer pH 3.0. *Ta*proA1 was pre-incubated for 30 min at the desired temperature ranges to evaluate the thermostability. The concentration of *Ta*proA1 used in all the assays was 1.73 mg/mL. The protease activity was performed using casein (1%, w/v) as a protein substrate.

### Effects of metal ions, chemical reagents, and inhibitors on *Ta*proA1 activity

A solution of *Ta*proA1 (1.73 mg/mL) was incubated with various metal ions (Ba^2+^, Ca^2+^, Co^2+^, Cr^3+^, Cu^2+^ Fe^2+^, Fe^3+^, Li^+^, Mg^2+^, Mn^2+^, Sn^2+^, Sr^2+^, and Zn^2+^), chemical reagents (EDTA, SDS, and Triton X-100), and protease inhibitors (Pepstatin A, iodoacetamide, PMSF, and EDTA) at 50 °C for 30 min, and then the residual protease activities were evaluated under the optimal conditions (pH 3.0, 50 °C). All the above assays were performed using casein (1%, w/v) as a protein substrate. The final concentration of chemical reagents and metal ions was 1 mM, and protease inhibitors were 0.01 mM–5 mM. A control without reagents was defined as 100% activity.

### Substrate specificity and cleavage sites of *Ta*proA1

The substrate specificity of *Ta*proA1 was determined using different protein substrates (1%, w/v) such as casein, myoglobin, hemoglobin, bovine serum albumin, albumin HAS, skimmed milk, albumin egg, whey protein, soy protein isolate, gelatin, azo-casein, β-lactoglobulin, protamine sulfate, and collagen under the optimal conditions (pH 3.0, 50 °C). The protease activity determined using casein was defined as the control (100%). The oxidized insulin B chain (0.1%, w/v) was mixed with *Ta*proA1 (5 U/mL) in 50 mM citrate buffer pH 3.0 for 1 and 12 h. An equal volume of 0.1% (v/v) trifluoroacetic acid was added to stop the reaction. To determine the cleavage sites, the mixtures were analyzed by MALDI-TOF/MS (AB Sciex 4800 plus, USA).

### Preparation of duck blood peptides by *Ta*proA1

Based on the high hydrolysis activity of *Ta*proA1 towards myoglobin and hemoglobin, the duck blood hemoglobin and plasma proteins were hydrolyzed by *Ta*proA1 (E/S: 1000 U/g, pH 3.0, 50 °C) for 3, 6, and 9 h. The hydrolysis reaction was stopped by heating in boiling water for 10 min to inactive *Ta*proA1. The hydrolysate supernatants were collected to further analyze the ACE inhibitory activity and molecular weight distribution. The protein recovery rate was evaluated by the Kjeldahl and Lowry methods (Lowry 1951). The degree of hydrolysis (DH) was determined by the o-phthaldialdehyde (OPA) assay (Nielsen et al. 2001), and the DH was calculated according to the following formula:

$$\textrm{DH}\ \left(\%\right)=\textrm{h}/{\textrm{h}}_{\textrm{tot}}\times 100\%$$

$$h=\frac{Serine{{NH}_2}-\beta}{\alpha}\textrm{meqv}/\textrm{g}\ \textrm{protein}$$

$$Serine{NH}_2=\frac{OD_{sample}-{OD}_{blank}}{OD_{standard}-{OD}_{sample}}\times 0.9516\textrm{meqv}/\textrm{L}\times \frac{0.1\times 100}{X\times P}$$

where *h* is the number of hydrolyzed bonds in the hydrolysates; *h*_tot_ is the total number of peptide bonds per protein equivalent; for duck blood, *α* and *β* are 1.0 and 0.4, respectively; *SerineNH*_2_ = meqv serine NH_2_/g protein; *X* is the mass of sample (g); and *P* is the protein content (%) in the hydrolysates.

A high-performance liquid chromatography (HPLC) system was utilized to analyze the molecular weight (Mw) distribution of the hydrolysates (Xie et al. 2014). The molecular weight distribution of the hydrolysates was divided into four fractions (< 1 kDa, 1–5 kDa, 5–10 kDa, and > 10 kDa).

The in vitro ACE inhibitory activity was determined according to the previous method (Cushman and Cheung 1971). Briefly, 20 μL of hydrolysate solution and 120 μL of substrate solution (5 mM N-Hippuryl-His-Leu hydrate in 0.1 M sodium borate buffer pH 8.3 containing 0.3 M NaCl) were incubated at 37 °C for 5 min. Then, 10 μL of ACE (0.1 U/mL) was added to start the reaction at 37 °C for 60 min. Next, 150 μL of 1 M HCl was added to stop the reaction. Hippuric acid was extracted by the addition of 1 mL ethyl acetate, and the mixture was centrifuged at 4000 rpm for 10 min. The supernatant (750 μL) was collected and dried in an oven at 105 °C for 30 min. The released hippuric acid was dissolved in 500 μL of deionized water, and the absorbance was measured at 228 nm. Sodium borate buffers pH 8.3 (0.1 M containing 5 mM N-Hippuryl-His-Leu hydrate and 0.3 M NaCl) without the hydrolysate and ACE were used as a control and as a blank, respectively. The following formula was used to calculate the in vitro ACE inhibitory activity:

$$\textrm{ACE}\ \textrm{inhibitory}\ \textrm{activity}\ \left(\%\right)=\left({A}_b-{A}_a\right)/\left( A_{b}- A_{c}\right)\times 100\%$$

where *A*_*a*_ represents the absorbance of the sample, *A*_*b*_ represents the absorbance of the control, and *A*_*c*_ represents the absorbance of the blank. The IC_50_ value (mg/mL) is defined as the concentration of hydrolysate that inhibited 50% of the ACE activity.

### Statistical analysis

The results were expressed as the mean ± standard deviation (SD). IBM SPSS 21.0 software (SPSS Inc., Chicago, IL, USA) was used to analyze all statistical data. One-way ANOVA indicated significant differences at *P* < 0.05, and Duncan’s multiple range tests were used.

## Results

### Bioinformation analysis of *Ta*proA1

A novel aspartic protease gene (*Tapro*A1) from *T. asperellum* was cloned and identified (GenBank no. GFP56020.1). The full-length gene was 1643 bp with an intron of 77 bp and an open reading frame of 1566 bp encoding 521 amino acid residues (Fig. S1). The isoelectric point (*pI*) and molecular weight of mature *Ta*proA1 were predicted to be 4.08 and 36 kDa, respectively. *Ta*proA1 showed 16 potential O-glycosylation sites and no N-glycosylation sites. It contained a predicted signal peptide sequence (20 aa), a pro-peptide sequence, and a mature catalytic domain sequence. Two highly conserved aspartic acid residues (Asp225 and Asp411) were located at the active site to play a catalytic role (Fig. S2).

**Fig. 1:**
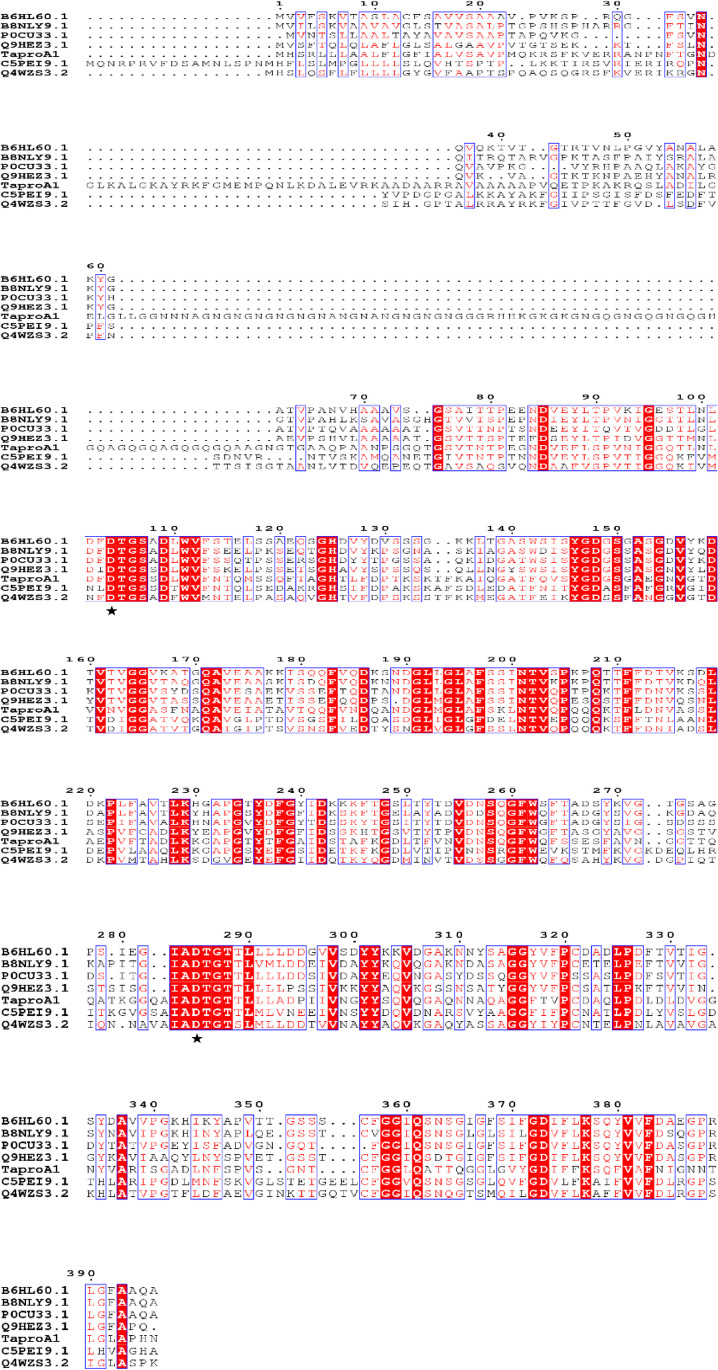
Multiple sequence alignments of *Ta*proA1 from *T. asperellum* with other A1 family aspartic proteases. NCBI GenBank accession numbers were as follows: Q9HEZ3.1 from *Penicillium janthinellum*, P0CU33.1 from *Aspergillus oryzae*, B6HL60.1 from *Penicillium rubens* Wisconsin 54-1255, Q4WZS3.2 from *Aspergillus fumigatus* Af293, C5PEI9.1 from *Coccidioides posadasii* C735, B8NLY9.1 from *Aspergillus flavus* NRRL3357. The strictly conserved characteristic motifs were highlighted with red boxes. The black asterisks indicate potential catalytic residues in the A1 family of aspartic proteases

Multiple sequence alignments revealed that *Ta*proA1 shared 52.8% sequence identity with the aspartic protease PEP3 from *Coccidioides posadasii* C735 (GenBank no. C5PEI9.1), followed by 50.7% sequence identity with aspartic protease PEPA from *Penicillium rubens Wisconsin* 54-1255 (GenBank no. B6HL60.1) (Fig. 1). A phylogenetic tree was constructed to identify the evolutionary relationship between *Ta*proA1 and other A1 family aspartic proteases (Fig. S3), suggesting that *Ta*proA1 belongs to the Aspergillopepsin I family containing the typical strictly conserved characteristic motif “DTGT/S”. *Ta*proA1 belongs to a new unknown branch with Q4WZS3 and is far from other A1 family proteases. These results indicated that *Ta*proA1 is a novel member of the A1 family aspartic protease.

### High-level production and purification of *Ta*proA1

**Fig. 2:**
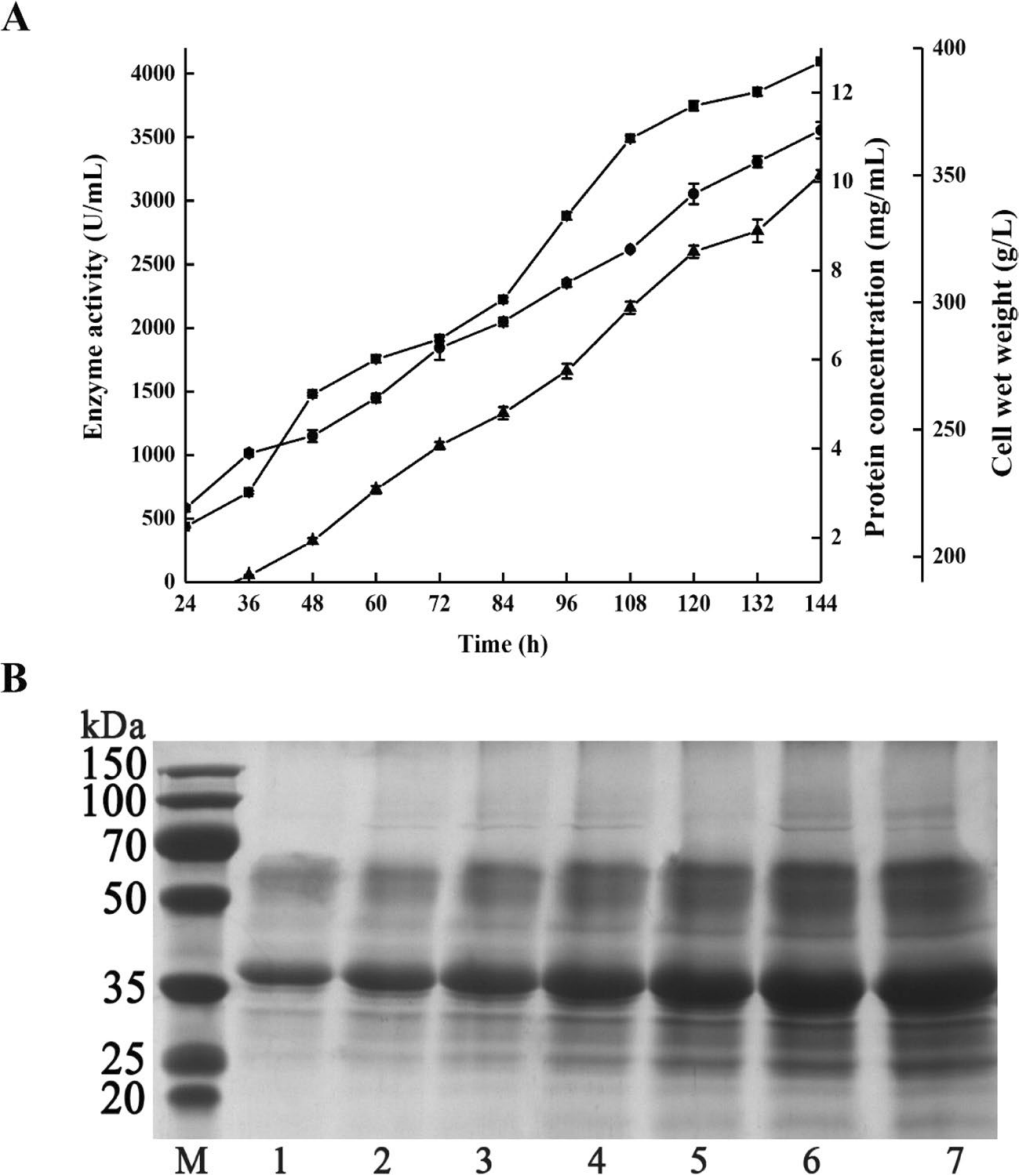
Time-course of high cell-density fermentation of *K. phaffii* in a 5 L fermenter (**A**) and SDS-PAGE analysis of the extracellular proteins (**B**). Enzyme activity (■), protein concentration (▲), and cell wet weight (●) were monitored per 12 h during the fermentation process. The enzyme activity was determined in 50 mM citrate buffer pH 3.0 at 50 °C using casein (1%, w/v) as the substrate. Lane M, standard protein molecular marker; Lanes 1–7, the fermentation supernatant was collected at 24, 48, 72, 96, 108, 120, and 144 h, respectively

**Fig. 3:**
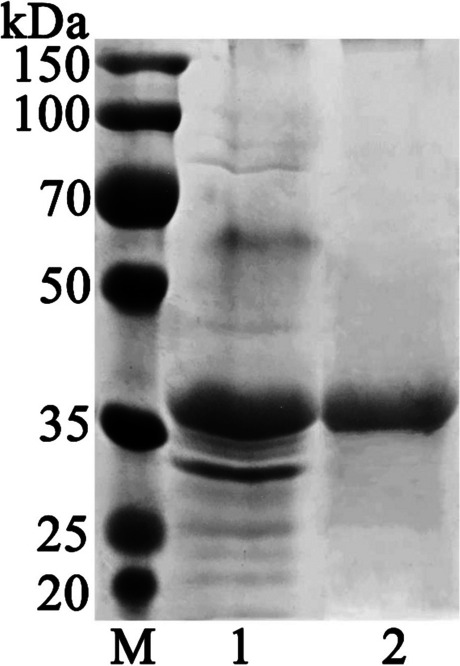
SDS-PAGE analysis of the proteins during purification of *Ta*proA1. Lane M, standard protein molecular marker; Lane 1: crude enzyme; Lane 2: purified enzyme

The recombinant strain with high protease activity screened by Geneticin G418 was subjected to fed-batch fermentation for the production of *Ta*proA1 in a 5 L fermenter. After 144 h, the protease activity, protein concentration, and cell wet weight were up to 4092 U/mL, 10.2 mg/mL, and 368 g/L, respectively (Fig. 2A). The protein concentration of *Ta*proA1 gradually increased, and a protein band of approximately 36 kDa was detected by SDS-PAGE during the fermentation process (Fig. 2B). *Ta*proA1 was purified to homogeneity by ion-exchange chromatography with a recovery yield of 52.8% and 1.7-fold purification (Fig. 3 and Table S2). The purification profile of *Ta*proA1 on the QSSF column is shown in Fig. S4. The specific activity of purified *Ta*proA1 was 685.0 U/mg towards casein (Table S2).

### Biochemical characterization of *Ta*proA1

**Fig. 4:**
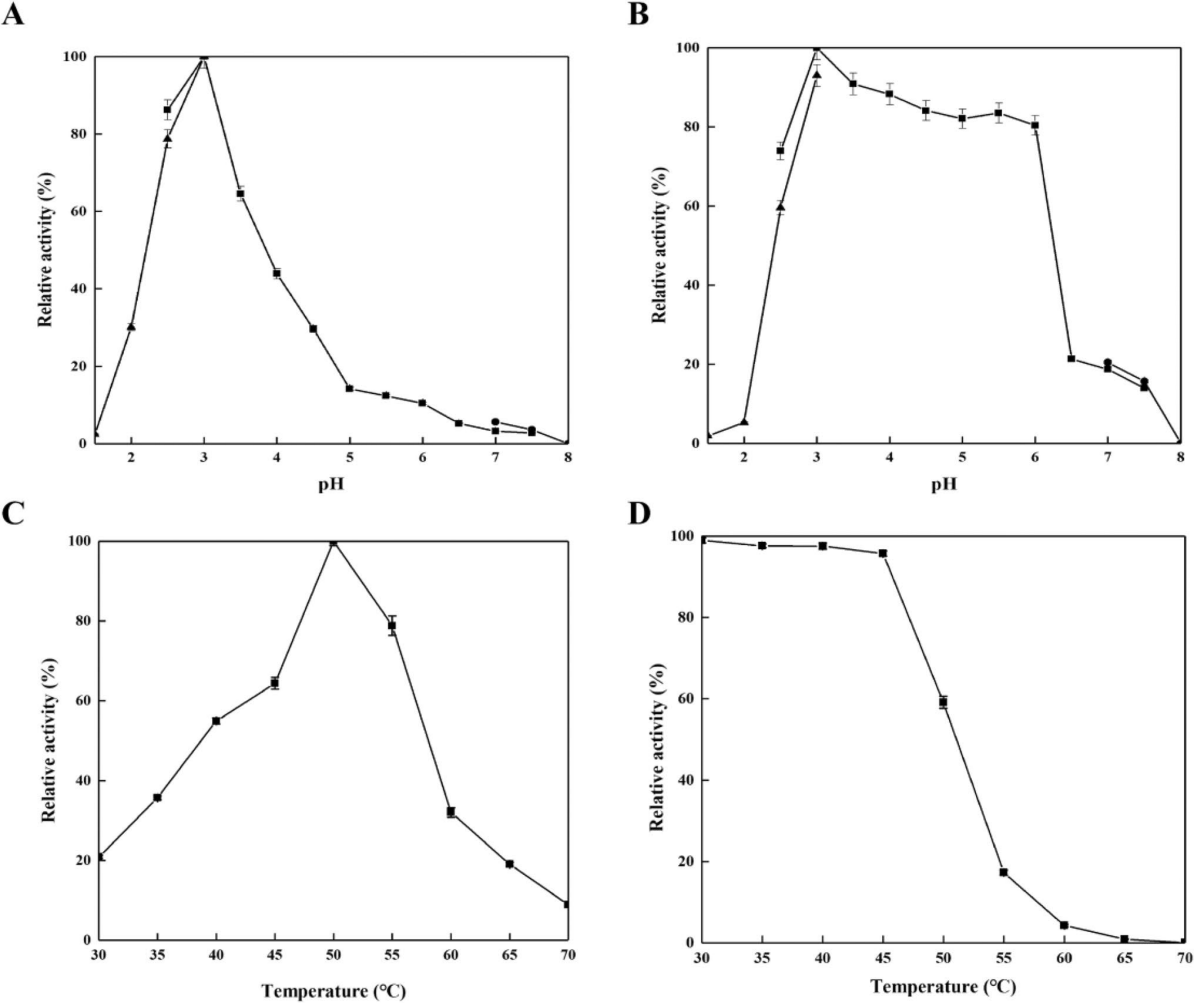
Optimal pH (**A**), pH stability (**B**), optimal temperature (**C**), and thermostability (**D**) of the purified *Ta*proA1. Buffers were used: glycine-HCl (▲), pH 1.5–3.0; citrate (■), pH 2.5–7.5; Tris-HCl (●), pH 7.0–8.0. The optimal pH of *Ta*proA1 was measured in above 50 mM different pH buffers at 40 °C. The pH stability of *Ta*proA1 was measured in the above-mentioned buffers at 40 °C for 30 min, and the residual activity was measured by the standard assay. The optimal temperature of *Ta*proA1 was determined at different temperatures (30–70 °C) in 50 mM citrate buffer pH 3.0. The thermostability of *Ta*proA1 was determined in 50 mM citrate buffer pH 3.0 at different temperatures (30–70 °C) for 30 min, and the residual activity was determined by the standard assay

*Ta*proA1 showed optimal activity at pH 3.0 (Fig. 4A), and more than 80% of its initial activity was retained in the pH ranges of 3.0–6.0 (Fig. 4B). The optimal temperature of *Ta*proA1 was 50 °C (Fig. 4C). *Ta*proA1 displayed good stability up to 45 °C, which more than 80% of its initial activity was retained (Fig. 4D). Cu^2+^ exhibited a promoting effect on protease activity, while Ba^2+^ had no effect. Cr^3+^, Fe^3+^, Fe^2+^, and Sr^2+^ inhibited protease activity by 11.9%, 13.3%, 17.7%, and 19.6%, respectively. Triton X-100 decreased the protease activity by 41.1%, whereas SDS completely inhibited the protease activity (Table S3). Pepstatin A (0.02 mM) completely inhibited its activity, indicating that *Ta*proA1 is an aspartic protease. EDTA and iodoacetamide had no significant effect on *Ta*proA1 activity, while PMSF slightly inhibited the enzyme activity (Table 1).

**Table 1:** Effects of protease inhibitors on *Ta*proA1 activity

Inhibitors	Concentration (mM)	Specific activity (U/mg) ^a^	Relative activity ^b^ (%)
Control	0	685.0±1.3	100
Pepstatin A	0.01	4.8±0.1	0.7±0.1
	0.02	0±0	0±0
PMSF	1	614.9±3.8	89.8±0.5
	5	480.1±6.2	70.1±0.9
EDTA	1	667.4±4.9	97.4±0.7
	5	663.8±6.9	96.9±1.0
Iodoacetamide	1	670.6±6.5	97.9±0.9
	5	652.8±4.1	95.3±0.6

^a^The protease activity was measured under optimal conditions. All data were mean values ± standard deviations of triplicate tests

^b^Without protease inhibitors, the specific activity was defined as 100%

### Substrate specificity and cleavage sites of *Ta*proA1

**Fig. 5:**
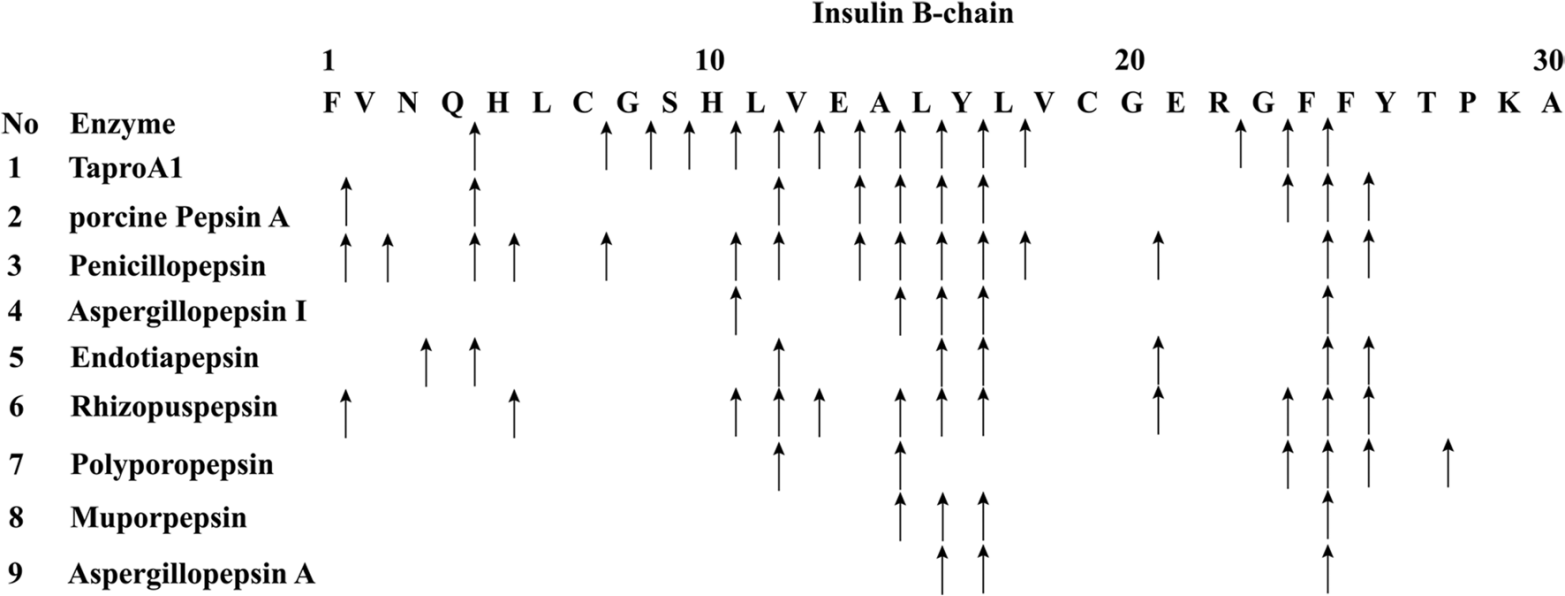
The cleavage sites of *Ta*proA1 on the oxidized insulin B chain. Other fungal aspartic proteases (porcine pepsin A, penicillopepsin, aspergillopepsin I, Endotiapepsin, rhizopuspepsin, polyporopepsin, muporpepsin, and aspergillopepsin A) cleavage sites are also shown for comparison. The arrows indicate the cleavage sites

The substrate specificity of *Ta*proA1 towards different protein substrates is shown in Table 2. *Ta*proA1 exhibited broad substrate specificity and excellent hydrolysis activity towards myoglobin (116.4%) and hemoglobin (113.5%), followed by bovine serum albumin (82.9%), albumin HAS (45.8%), skimmed milk (36.4%), albumin egg (25.7%), whey protein (12.9%), soy isolate protein (7.8%), gelatin (1.6%), and azo-casein (1.2%). In contrast, *Ta*proA1 did not hydrolyze β-lactoglobulin, protamine sulfate, and collagen. Furthermore, *Ta*proA1 spliced the oxidized insulin B-chain at 15 bonds (H4-Q5, C7-G8, G8-S9, S9-H10, H10-11L, 11L-12V, 12V-13E, 13E-14A, 14A-15 L, 15 L-16Y, 16Y-17L, 17L-18V, 22R-23G, 23G-24F, and 24F-25F) (Fig. 5).

**Table 2:** Substrate specificity of *Ta*proA1

Substrates	Specific activity (U/mg) ^a^	Relative activity ^b^ (%)
Casein	685.0±1.3	100
Myoglobin	797.3±9.0	116.4±1.3
Hemoglobin	777.8±4.3	113.5±0.6
Bovine serum albumin	568.3±5.9	82.9±0.9
Albumin HAS	313.4±5.9	45.8±0.9
Skimmed milk	249.3±4.9	36.4±0.7
Albumin egg	175.9±2.3	25.7±0.3
Whey protein	88.4±3.5	12.9±0.5
Soy isolated protein	53.7±1.6	7.8±0.2
Gelatin	11.0±0.2	1.6±0.1
Azo-casein	8.3±2.1	1.2±0.3
β-lactoglobulin	----- ^c^	----- ^c^
Protamine sulfate	-----^c^	----- ^c^
Collagen	----- ^c^	----- ^c^

^a^The protease activity was measured under optimal conditions. All data were mean values ± standard deviations of triplicate tests

^b^The protease activity towards casein was defined as 100%

^c^Not activity detected

### Preparation of duck blood peptides by *Ta*proA1

**Fig. 6:**
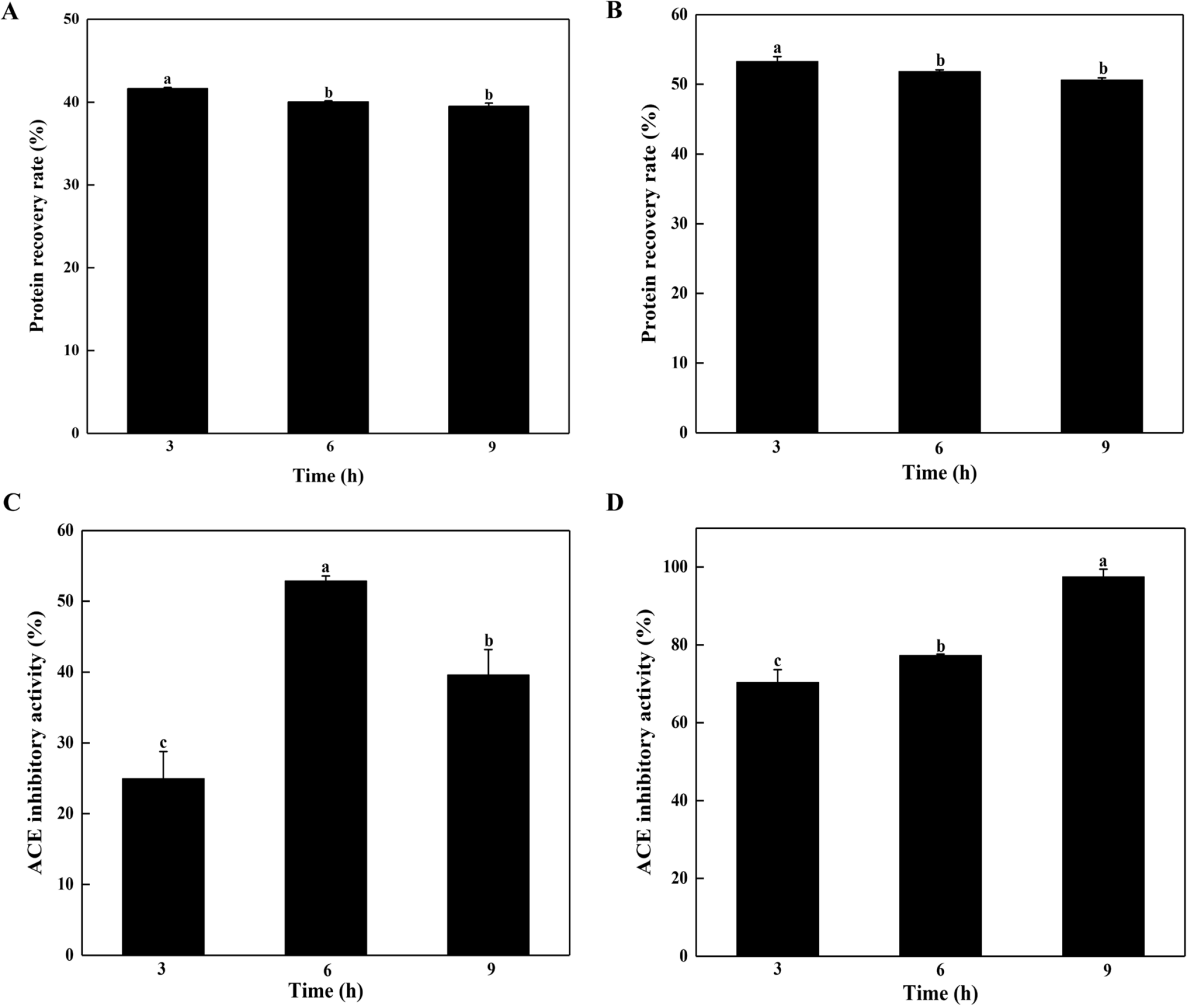
Determination of protein recovery rate from duck blood hemoglobin (**A**) and plasma proteins (**B**) hydrolysates. The determination of ACE inhibitory activity from duck blood hemoglobin (**C**) and plasma proteins (**D**) hydrolysates at a protein concentration of 0.1 mg/mL. The different lowercase letters represent significant differences (*P* <0.05)

The ACE inhibitory activity of duck blood proteins hydrolysates was analyzed. When plasma protein and hemoglobin from duck blood were hydrolyzed for 3 h, the protein recovery rates were 52.3% and 41.6%, respectively (Fig. 6A, B). There was no significant difference among the DH of hemoglobin and plasma proteins at 3 and 6 h, respectively. The DH of hemoglobin and plasma proteins was 59.5% and 54.0%, respectively, which reached a maximum at 9 h (Fig. S5). As shown in Fig. S6 and Table 3, the molecular weight distribution of the duck plasma protein hydrolysate changed from 73.2% (Mw >10 kDa) to small peptides with Mw < 1 kDa (82.1%) at 9 h. For the duck hemoglobin hydrolysate, the molecular weight distribution changed from 75.8% (Mw > 10 kDa) to small peptides with Mw < 1 kDa (82.6%) at 6 h. The duck plasma protein hydrolysate exhibited the highest ACE inhibitory activity of 97.9% at 9 h, and the duck hemoglobin hydrolysate exhibited the highest ACE inhibitory activity of 52.9% at 6 h (Fig. 6C, D). The IC_50_ values of duck plasma protein and hemoglobin hydrolysates were 0.091 mg/mL and 0.105 mg/mL, respectively.

**Table 3:** The molecular weight distribution and IC_50_ values of duck blood proteins hydrolysate

Sample	Hydrolysis time (h)	Molecular weight distribution ^a^ (%)				IC_50_ (mg/mL)
		<1 kDa	1–5 kDa	5–10 kDa	>10 kDa	
Hemoglobin	0	7.77	5.00	11.48	75.75	0.105
	3	79.05	20.57	0.05	0.32	
	6	82.55	17.14	0.03	0.27	
	9	84.36	15.57	0.03	0.03	
Plasma protein	0	18.08	4.51	4.21	73.20	0.091
	3	75.19	24.40	0.01	0.38	
	6	77.43	22.26	0.01	0.29	
	9	82.09	17.69	0.00	0.20	

^a^The molecular weight distribution of hydrolysates was determined by HPLC at the protein concentration of 1.0 mg/mL

## Discussion

In this study, a novel aspartic protease gene (*Tapro*A1) from *T. asperellum* was successfully mined and expressed in *K. phaffii.* The pro-peptide sequence plays a vital role in the folding and secretion of active proteases. It is automatically removed by self-cleavage during the maturation process (Demidyuk et al. 2010; Peng et al. 2021). The band size of expressed target protein suggested that *Ta*proA1 was secreted as a mature enzyme through autocatalytic activation (Fig. 2B). The neutral metalloproteinase NPI from *A. oryzae* and the alkaline serine protease SPTK from *Trichoderma koningii* were efficiently expressed in *K. phaffii*, and the protease activities were 43101 U/ml and 15900 U/ml, respectively (Ke et al. 2012; Shu et al. 2016). However, the expression levels of acid proteases in *K. phaffii*, such as RmproA (3480.4 U/mL) from *Rhizomucor miehei* CAU432 (Sun et al. 2018), Apa1 (1500 U/mL) from *A. niger* (Wei et al. 2023), MCAP (410 MCU/mL, rennet activity) from *Mucor circinelloides* (Kangwa et al. 2018), PsAPA (89.3 U/mL) from *Penicillium* sp. XT7 (Guo et al. 2021), TAlP (67.8 U/mL) from *Talaromyces leycettanus* JCM12802 (Guo et al. 2019), and TAASP (18.5 U/mL) from *Trichoderma asperellum* (Yang et al. 2013) are relatively low (Table S4). Here, the expression level of *Ta*proA1 (4092 U/mL) was significantly higher than those of most aspartic proteases produced in *K. phaffii* (Table S4). Therefore, the high-level expression of *Ta*proA1 should be beneficial for potential applications.

*Ta*proA1 was purified to homogeneity by QSSF with a recovery yield of 52.8% (Table S2). Compared with other proteases, the purification efficiency of *Ta*proA1 was higher than that of the aspartic proteases RmproA (16.8%) (Sun et al. 2018) and RmproB (18.8%) (Wang et al. 2021) but lower than that of the serine protease FgAPT4 (59.6%) (Wang et al. 2023) and the aspartic protease Apa1 (72%) (Wei et al. 2023). Generally, aspartic proteases have optimal pH and pH stability under acidic conditions (Table S4). The optimal pH of *Ta*proA1 (Fig. 4A) is consistent with that of TlAP from *Talaromyces leycettanus* JCM12802 (Guo et al. 2019), lower than those of TAASP (pH 4.0) from *Trichoderma asperellum* (Yang et al. 2013) and RmproA (pH 5.5) from *R. miehei* CAU432 (Sun et al. 2018) but higher than those of PepAb (pH 2.5) from *A. niger* (Song et al. 2020) and RmproB (pH 2.5) from *R. miehei* CAU432 (Wang et al. 2021). The optimal temperature of *Ta*proA1 (Fig. 4C) was the same as those of PepA, PepAb, and PepAc from *A. niger* (Song et al. 2020) and PepA from *A. oryzae* (Yue et al. 2019) but lower than those of TlAP (55 °C) from *Talaromyces leycettanus* JCM12802 (Guo et al. 2019) and RmproA (55 °C) from *R. miehei* CAU432 (Sun et al. 2018). *Ta*proA1 was stable up to 45 °C (Fig. 4D) and retained almost all its initial activity after incubation for 30 min. The thermostability of *Ta*proA1 (59.2%) was higher than that of *Ps*APA (0%) from *Penicillium* sp. XT7 (Guo et al. 2021) and rP6281 (almost 0%) from *Trichoderma harzianum* (Deng et al. 2018) at 50 °C for 30 min. Additionally, Cu^2+^ effectively enhanced the activity of aspartic proteases in other studies (Deng et al. 2018; Guo et al. 2019). SDS completely inhibited protease activity, which may be attributed to the denaturation of *Ta*proA1 (Sun et al. 2018). Most aspartic proteases exhibited the highest hydrolysis activity towards casein (Azadi et al. 2022; Guo et al. 2021; Sun et al. 2018; Wang et al. 2021). In this study, *Ta*proA1 showed the highest hydrolysis activity towards hemoglobin, followed by myoglobin and casein (Table 2). Microbial aspartic proteases preferentially cleave peptide bonds between hydrophobic or aromatic amino acid residues at the ends of protein substrates, such as Phe-Phe, Phe-Tyr, and Leu-Tyr. The hydrolysis specificity of *Ta*proA1 was closely related to the mode of cleavage of the substrate (Gao et al. 2018; Rao et al. 2011). Compared with other aspartic proteases, *Ta*proA1 showed different substrate cleavage patterns (Fig. 5). A mammalian aspartic protease (porcine pepsin A) showed a broad specificity of cleavage sites at L11-V12, E13-A14, A14-L15, L15-Y16, Y16-L17, F24-F25, and F25-Y26 of the oxidized insulin B chain (Rao et al. 2011). The specificity of *Ta*proA1 was similar to that of mammalian aspartic proteases. In addition, aspartic proteases have high affinity for the F24-F25 bond of the oxidized insulin B chain (Fig. 5), and pepsin-like aspartic proteases usually have higher substrate hydrolysis activity than chymosin-like aspartic proteases to degrade protein substrates into small peptides (Takyu et al. 2022).

Generally, the bioactivity of protein hydrolysates mainly depends on protein structure, the protease used, and the hydrolysis conditions. As shown in Fig. S7, the activity of *Ta*proA1 decreased with the extension of hydrolysis time during the preparation of duck blood peptides. According to the IC_50_ values, duck plasma protein was more suitable for efficient hydrolysis by *Ta*proA1 than hemoglobin to prepare bioactive peptides with high ACE inhibitory activity (Table 3). Microbial proteases have been widely used for the preparation of bioactive peptides. Two aspartic proteases, RmproA and RmproB, from *R. miehei* CAU432 were used to produce peptides with ACE inhibitory activity from turtle meat and duck hemoglobin, respectively. When the protein concentration was 1.0 mg/mL, turtle meat hydrolyzed by RmproA showed 88% ACE inhibitory activity (Sun et al. 2018). When the protein concentration was 0.5 mg/mL, duck hemoglobin hydrolyzed by RmproB showed 90.7% ACE inhibitory activity (Wang et al. 2021). In this study, when the protein concentration of the hydrolysates was 0.1 mg/mL, duck hemoglobin and plasm protein hydrolysates showed excellent ACE inhibitory activities of 52.88% and 97.88%, respectively (Fig. 6A, B). Duck hemoglobin hydrolysate by *Ta*proA1 with ACE inhibitory activity has a lower IC_50_ value than duck hemoglobin hydrolysate by RmproB (Wang et al. 2021). This result indicated that *Ta*proA1 showed a better effect than that of RmproB in preparing ACE inhibitory peptides from duck hemoglobin. Currently, the commercial aspartic protease pepsin has been applied to prepare bioactive peptides from blood proteins. ACE inhibitory peptides were prepared by hydrolyzing porcine hemoglobin and bovine plasma proteins with pepsin, and the IC_50_ values were 1.53 mg/mL and 17.19 mg/mL, respectively (Deng et al. 2014; Hyun and Shin 2000). These results indicated that *Ta*proA1 has great potential in preparing peptides with ACE inhibitory activity from duck blood proteins.

In conclusion, a novel aspartic protease (*Ta*proA1) from *T. asperellum* was successfully expressed in *K. phaffii* GS115. It was efficiently produced by fed-batch fermentation in a 5 L fermenter and yielded a protease activity of 4092 U/mL. *Ta*proA1 showed optimal activity at pH 3.0 and 50 °C, a broad substrate specificity, and the highest hydrolysis activity towards myoglobin and hemoglobin. Moreover, duck blood proteins were efficiently hydrolyzed by *Ta*proA1 to prepare duck blood peptides with high ACE inhibitory activity, showing IC_50_ values of 0.105 mg/mL and 0.091 mg/mL for hemoglobin and plasma protein hydrolysates, respectively. The high-level expression and unique properties of *Ta*proA1 make it great value for the production of bioactive peptides.

## Supplementary information

ESM 1 (PDF 1124 kb)

## Data Availability

All data generated or analyzed in this study are included in this published article and its supplementary information files.
