# Supplementary material for: Characterization of a novel aspartic protease from Trichoderma asperellum for the preparation of duck blood peptides
Source: Appl Microbiol Biotechnol. 2024 Jan 13;108:131. doi: 10.1007/s00253-023-12848-y (PMC10787677; doi:10.1007/s00253-023-12848-y)
Supplement: Supplementary file 1 [file 253_2023_12848_MOESM1_ESM.pdf]

**Supplementary materials**

# Characterization of a novel aspartic protease from *Trichoderma asperellum* for the preparation of duck blood peptides

**Yibin Xue [a,#], Qiaojuan Yan [b,#], Xue Li [b], Zhengqiang Jiang [a,c*]**

[a] Key Laboratory of Food Bioengineering (China National Light Industry), College of Food Science & Nutritional Engineering, China Agricultural University, Beijing 100083, China

[b] College of Engineering, China Agricultural University, Beijing 100083, China

[c] Food Laboratory of Zhongyuan, Luohe City, Henan Province 462000, China

[#] Yibin XUE and Qiaojuan YAN contribute equally to this work.

* Corresponding authors. Tel.: +86 10 62737689; fax: +86 10 82388508. E-mail: zhqjiang@cau.edu.cn

**Running title: a novel aspartic protease from *Trichoderma asperellum***

```
1       ATGCATTCACGATTACTTTTGGCGGCCCTTTTCTTGGGCTTCATTGCCCTGGTTTCGGCCGTCCCTATGCAGAAGCGTTCGTTCAAGGTG
        M  H  S  R  L  L  L  A  A  L  F  L  G  F  I  A  L  V  S  A  V  P  M  Q  K  R  S  F  K  V
91      GAGCGAAGAGCCAACCCCAACTTCACTGGAAACGATGGATTGAAGGCTCTGGGAAAGGCCTACCGCAAGTTCGGTATGGAGATGCCCCAG
        E  R  R  A  N  P  N  F  T  G  N  D  G  L  K  A  L  G  K  A  Y  R  K  F  G  M  E  M  P  Q
181     AACTTGAAGGATGCTCTTGAGGTTCGAAAGGCTGCCGACGCCGCCCGCCGAGCTGTTGCTGCCGCCGCCGCCCCTGTTCAGGAAACCCCC
        N  L  K  D  A  L  E  V  R  K  A  A  D  A  A  R  R  A  V  A  A  A  A  A  P  V  Q  E  T  P
271     AAGGCGAAGCGCCAGAGCCTTGCCGATATCCTCGGTGAGCTTGGCCTTCTTGGTGGCAACAACAACGCTGGAAATGGAAATGGAAATGGA
        K  A  K  R  Q  S  L  A  D  I  L  G  E  L  G  L  L  G  G  N  N  N  A  G  N  G  N  G  N  G
361     AACGGAAACGGAAACGCAAATGGAAATGCAAATGGAAATGGAAACGGAAACGGTGGTGGAAGGCACCATAAGGGCAAGGGTAAGGGCAAC
        N  G  N  G  N  A  N  G  N  A  N  G  N  G  N  G  N  G  G  G  R  H  H  K  G  K  G  K  G  N
451     GGTCAGGGCAACGGTCAGGGCAACGGTCAGGGCCACGGTCAAGCTGGTCAGGGCCAAGCTGGTCAAGGTCAGGGTCAAGCTGCTGGAAAC
        G  Q  G  N  G  Q  G  N  G  Q  G  H  G  Q  A  G  Q  G  Q  A  G  Q  G  Q  G  Q  A  A  G  N
541     GGAACTGGTGCTGCTCAGCCCGCTGCGAACCCCTCTGGTCAAACTGGAAGCGTCACCAACACCCCGGAAGGTAACGATGTTGAGTTCCTG
        G  T  G  A  A  Q  P  A  A  N  P  S  G  Q  T  G  S  V  T  N  T  P  E  G  N  D  V  E  F  L
631     TCCCCTGTCAACATTGGTGGCCAGACTCTGAACCTCGACTTCGACACTGGCTCTTCTGATCTCTGGGTGTTCAACACTCAAATGTCCTCT
        S  P  V  N  I  G  G  Q  T  L  N  L  D  F  D  T  G  S  S  D  L  W  V  F  N  T  Q  M  S  S
721     CAATTCACCGCCGGCCACACTCTCTTCGATCCCACCAAGAGCAAGACCTTCAAGGCTATCCAGGGTGCTACTTTCCAGGTCTCATACGGT
        Q  F  T  A  G  H  T  L  F  D  P  T  K  S  K  T  F  K  A  I  Q  G  A  T  F  Q  V  S  Y  G
811     GACGGCTCCGGTGCTGAGGGTAACGTCGGTACTGATGTTGTCAACGTTGGCGGTGCCTCCTTCAATGCCCAGGCTGTTGAGATTGCTACC
        D  G  S  G  A  E  G  N  V  G  T  D  V  V  N  V  G  G  A  S  F  N  A  Q  A  V  E  I  A  T
901     GCTGTTACTCAGCAATTCGTCAATGATCAGGCCAACGATGGTCTGATGGGTCTTGCCTTCTCTAAGCTTAACACCGTCCAGCCCCAGCAG
        A  V  T  Q  Q  F  V  N  D  Q  A  N  D  G  L  M  G  L  A  F  S  K  L  N  T  V  Q  P  Q  Q
991     CAGAAGACTTTCCTCGACAACGTCGCCAGCTCTCTCGCTGAGCCCGTGTTCACGGCCGATCTCAAGAAGGGTGCTCCCGGAACCTACACT
        Q  K  T  F  L  D  N  V  A  S  S  L  A  E  P  V  F  T  A  D  L  K  K  G  A  P  G  T  Y  T
1081    TTCGGTGCCATCGACTCCACCGCTTTCAAGGGTGATCTCACATTCGTCAATGTCGACAACAGCCAGGGCTTCTGGCAGTTCAGCAGCGAG
        F  G  A  I  D  S  T  A  F  K  G  D  L  T  F  V  N  V  D  N  S  Q  G  F  W  Q  F  S  S  E
1171    TCCTTTGCCGTCAACGGTGGCACTACCCAGCAGGCTACCAAGGGTGGCCAGGCCATCGCTGATACCGGCACCACTCTCCTACTTGCCGAC
        S  F  A  V  N  G  G  T  T  Q  Q  A  T  K  G  G  Q  A  I  A  D  T  G  T  T  L  L  L  A  D
1261    CCCATCATCGTCAACGGCTACTACTCCCAGGTTCAGGGTGCCCAGAACAACGCTCAGGCTGGTGGTTTCACTGTCCCCTGCGATGCTCAG
        P  I  I  V  N  G  Y  Y  S  Q  V  Q  G  A  Q  N  N  A  Q  A  G  G  F  T  V  P  C  D  A  Q
1351    CTTCCCGATCTTGACCTTGACGTTGGTGGCAACTACGTCGCCCGCATTAGCGGTGCCGATCTCAACTTCTCGCCTGTTTCTGGAAACACT
        L  P  D  L  D  L  D  V  G  G  N  Y  V  A  R  I  S  G  A  D  L  N  F  S  P  V  S  G  N  T
1441    TGCTTCGGCGGTCTCCAGGCTACTACGCAAGGTGGACTGGGTGTCTACGGTGACATCTTCTTCAAGTCGCAGTTTGTGGCTTTCAACATT
        C  F  G  G  L  Q  A  T  T  Q  G  G  L  G  V  Y  G  D  I  F  F  K  S  Q  F  V  A  F  N  I
1531    GGCAACAACACCCTGGGATTGGCTCCTCACAACTAA
        G  N  N  T  L  G  L  A  P  H  N  *
```

**Fig. S1** Nucleotide and deduced amino acid sequences of the aspartic protease gene (*Tapro*A1) from *T. asperellum.* The start and stop codons were boxed. The signal peptide was marked by the underline. Putative potential O- and N-linked glycosylation sites were displayed on the gray background and boxed, respectively. The translation termination was marked with an asterisk.

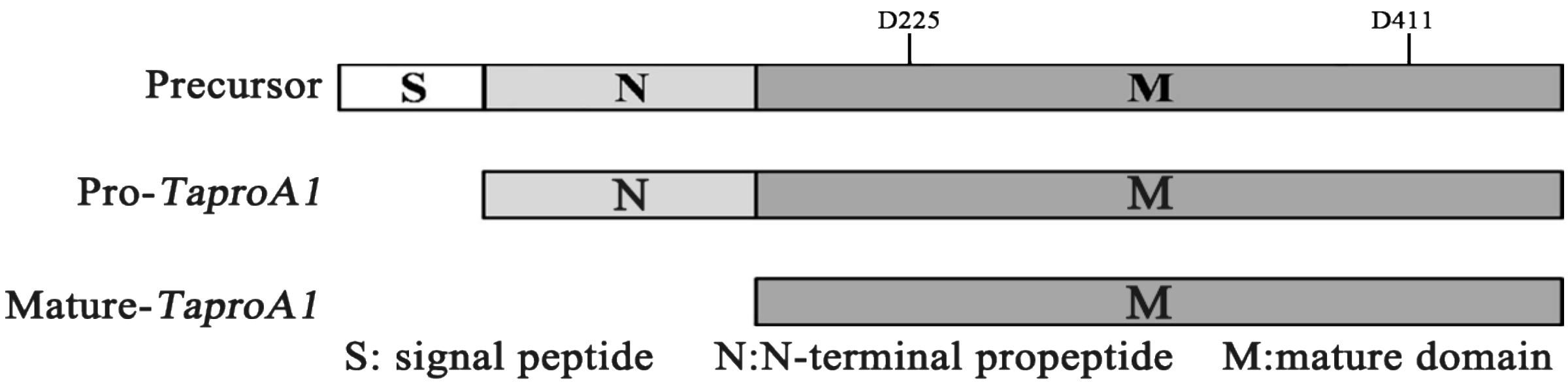


**Fig. S2** Schematic spectrum of the aspartic protease (*Ta*proA1) precursor. The putative *Ta*proA1 consists of a signal peptide sequence (S), a pro-peptide sequence (N), and a putative mature catalytic domain (M). Two catalysis residues D225 and D411 were marked in the protein sequence.

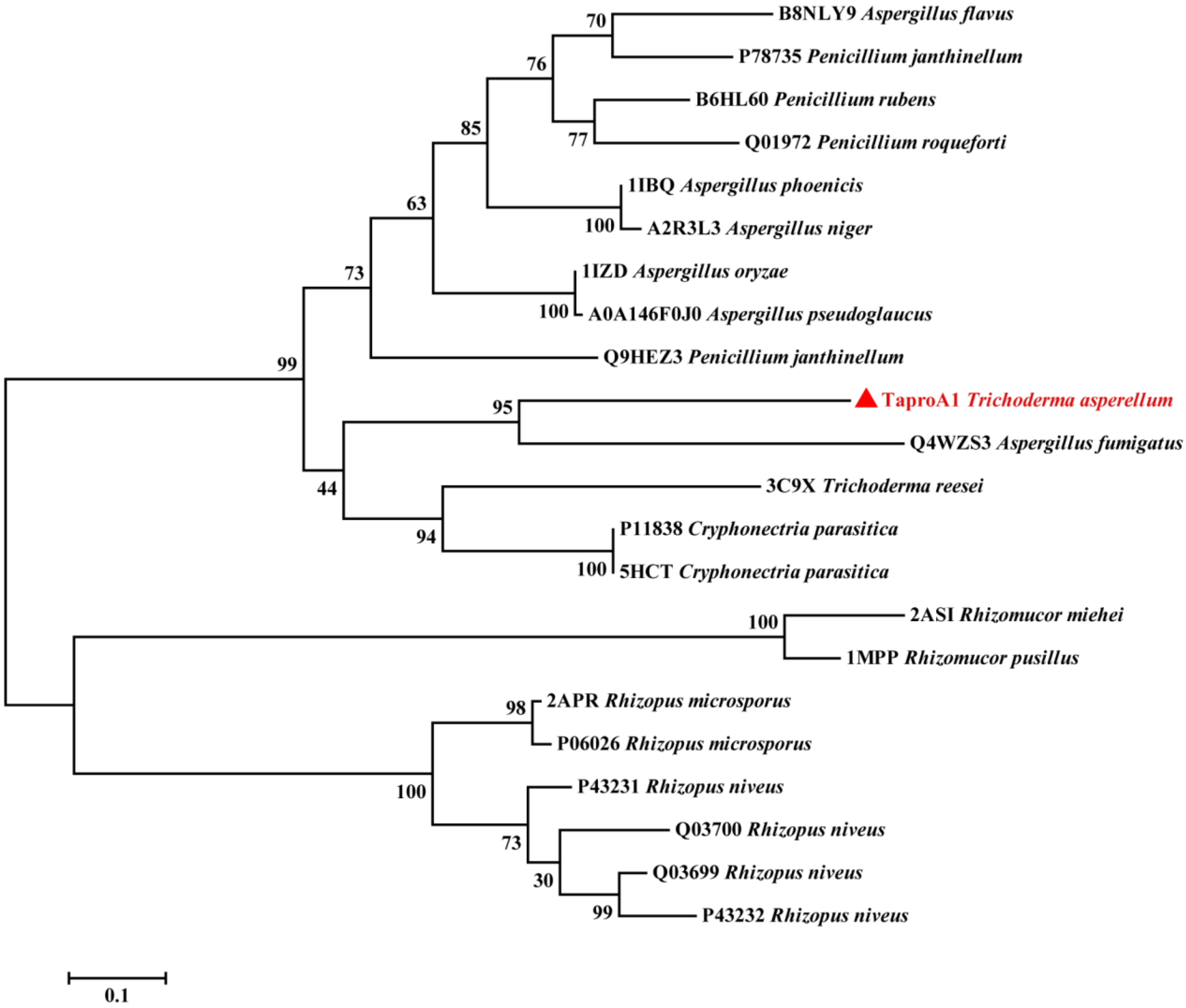


**Fig. S3** Phylogenetic tree analysis of *Ta*proA1 and other aspartic proteases from the A1 family members. *Ta*proA1 (GenBank accession number: GFP56020.1) was used as the query sequence, and the amino acid sequences of other aspartic proteases from the A1 family were obtained by BLAST analysis. The maximum likelihood method was used to construct the evolutionary tree. These sequences of host fungi were labeled with their GenBank accession number or PDB ID. The numbers indicated in the tree branches are the bootstrap values (%) based on 1000 replications. *Ta*proA1 was marked by a red triangle.

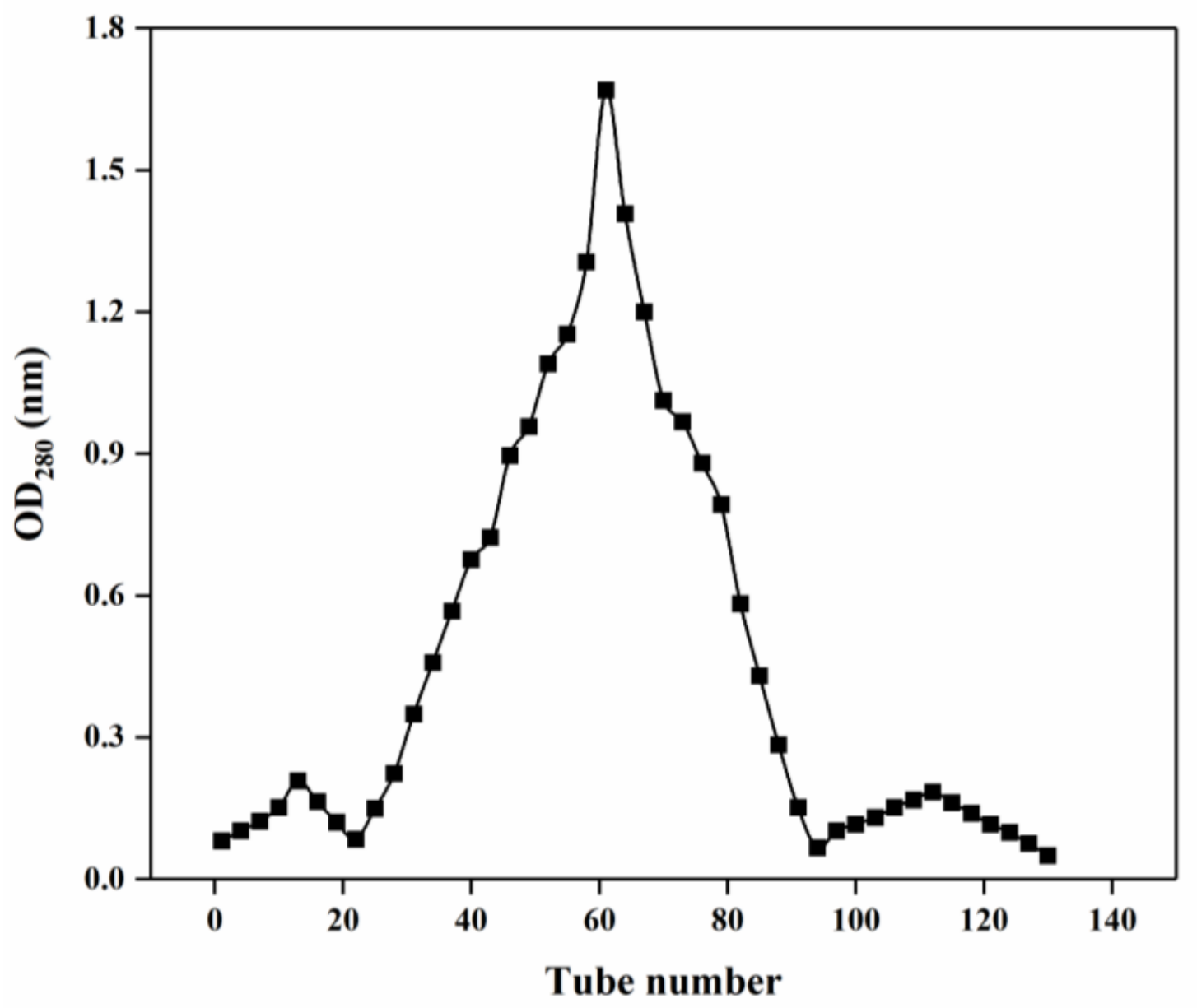


**Fig. S4** The elution profile of *Ta*proA1 on a Q Sepharose Fast Flow column pre-equilibrated with 20 mM phosphate buffer pH 6.0 and eluted using the same buffer in a linear gradient from 0 to 500 mM NaCl at a flow rate of 1.0 mL/min. The absorbance value of the elution sample was determined at 280 nm.

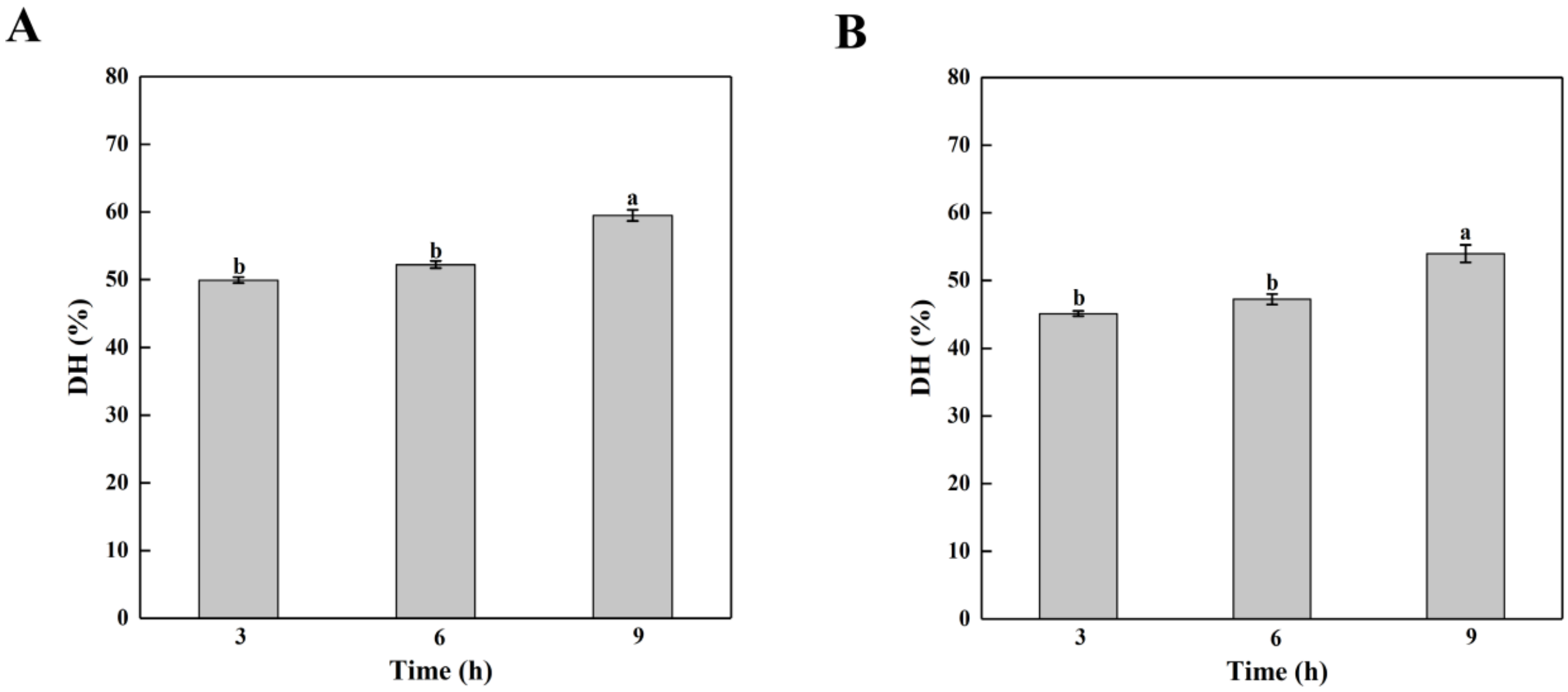


**Fig. S5** Degree of hydrolysis (DH) of duck blood hemoglobin (A) and plasma protein (B) hydrolyzed by *Ta*proA1 at different hydrolysis times was determined by the o-phthaldialdehyde (OPA) method. The different lowercase letters represent significant differences ($P < 0.05$).

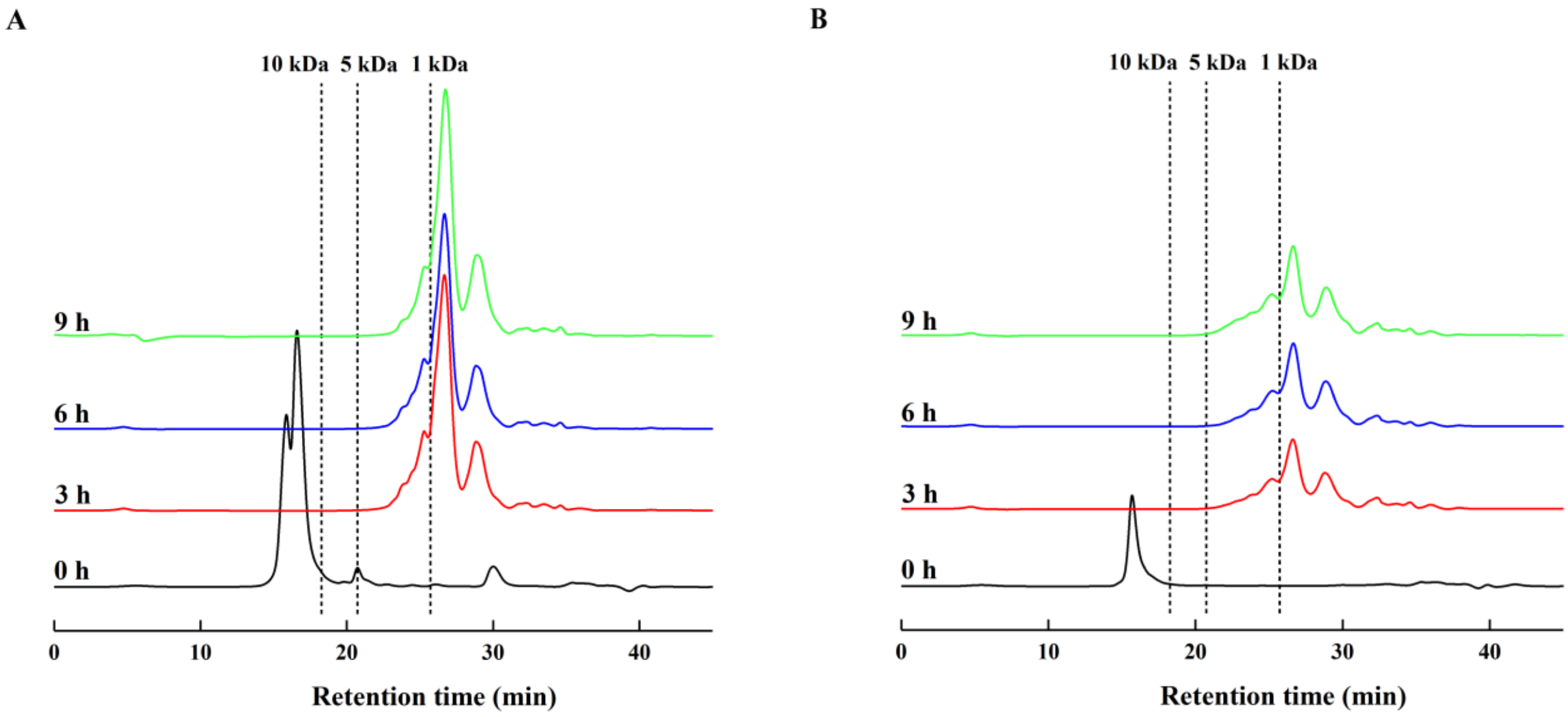


**Fig. S6** Molecular weight distribution of the duck blood hemoglobin (A) and plasma protein (B) hydrolysates produced by *Ta*proA1 at different hydrolysis times was determined by the HPLC and divided into four fractions (< 1 kDa, 1-5 kDa, 5-10 kDa, and > 10 kDa).

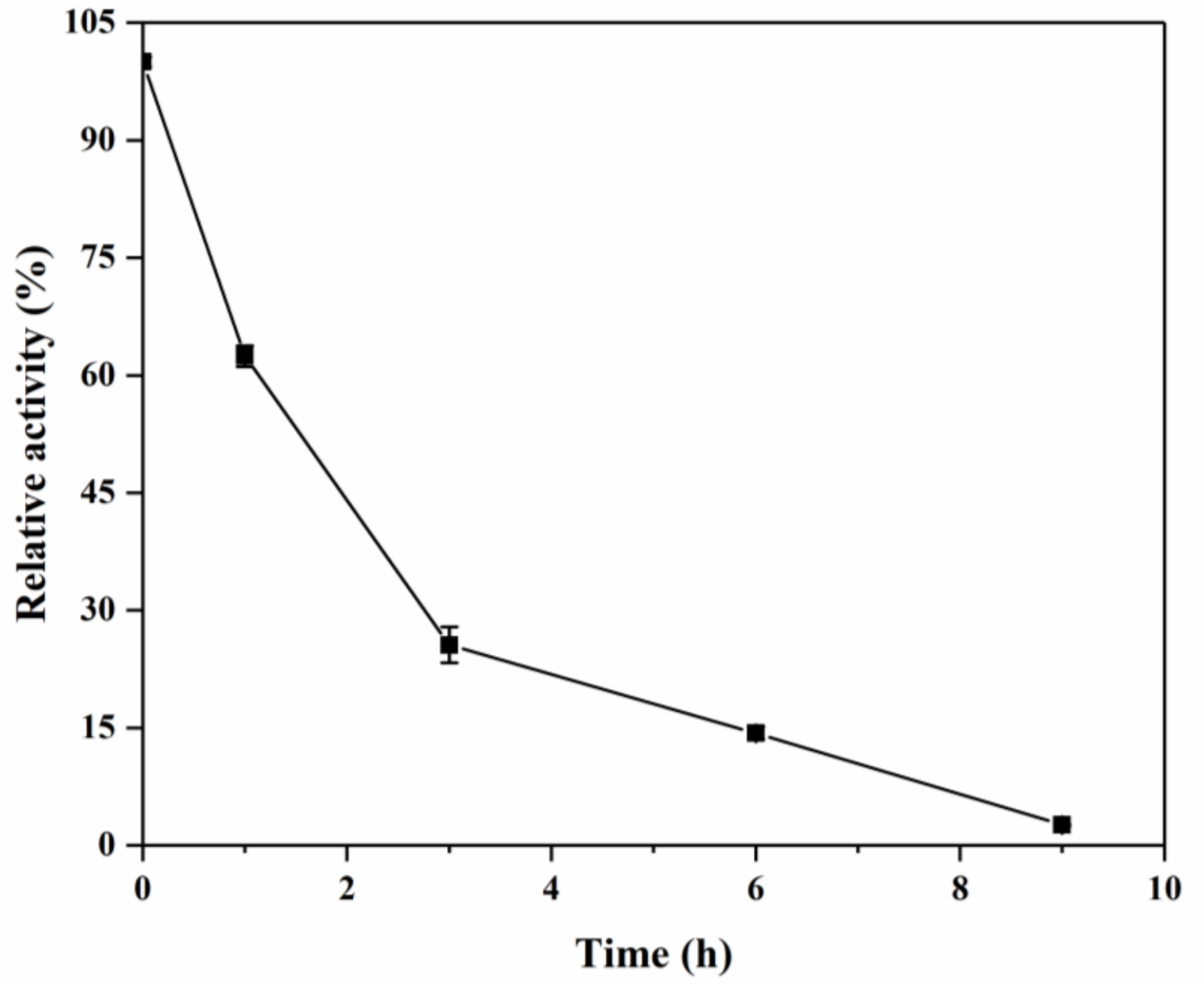


**Fig. S7** Thermostability evaluation of *Ta*proA1 at 50 °C for 1, 3, 6, and 9 h, respectively. The residual activity was determined using casein (1%, w/v) as substrate under the optimal reaction conditions (citrate pH 3.0, 50 °C). The concentration of *Ta*proA1 was 0.24 mg/mL in reaction conditions.

**Table S1** Primers used in this study

| Primer name [a] | Sequences (5´ ⟶ 3´) [b] |
|---|---|
| *Tapro*A1-F | GCTGAAGCTTACGTAGAATTCGTCCCTATGCAGAAGCGTTCG |
| *Tapro*A1-R | AGGCGAATTAATTCGCGGCCGCTTAGTTGTGAGGAGCCAATCCCAG |
| 5´AOX1 | GACTGGTTCCAATTGACAAGC |
| 3´AOX1 | GGCAAATGGCATTCTGACATCC |

a: The primer pair *Tapro*A1-F/R was used to amplify the *Tapro*A1 gene. The 5´ AOX1 and 3´ AOX1 primers were used to confirm whether the *Tapro*A1 gene was integrated into the *K. phaffii* GS115 genome.

b: Dotted line, restriction sites (*Eco*RI and *Not*I); solid line, homologous bases.

**Table S2** Purification summary of *Ta*proA1

| Purification step | Total activity (U)[a] | Total protein (mg)[b] | Specific activity (U $mg^{-1}$)[c] | Purification factor (-fold)[d] | Recovery (%)[e] |
|---|---|---|---|---|---|
| Crude enzyme | 122802.7 | 305.1 | 402.5 | 1.0 | 100 |
| QSFF | 64795.5 | 94.6 | 685.0 | 1.7 | 52.8 |

a: The enzyme activity was determined using casein (1%, w/v) as substrate at pH 3.0 and 50 °C.

b: The protein concentration was determined by the Lowry method using BSA as the standard.

c: The specific activity was defined as the enzyme activity per milligram of protein (U/mg).

d: The purification factor (-fold) was defined as the ratio of specific activity before and after purification.

e: The recovery (%) was calculated as the ratio of total activity before and after purification.

**Table S3** Effects of metal ions and chemicals on the activity of *Ta*proA1[a]

| Metal ions and reagents (1 mM) | Specific activity (U/mg) | Relative activity [b] (%) |
|---|---|---|
| Control | 685.0±1.3 | 100.0 |
| $Ba^{2+}$ | 687.1±1.5 | 100.3±0.2 |
| $Ca^{2+}$ | 676.1±3.7 | 98.7±0.5 |
| $Co^{2+}$ | 648.7±5.6 | 94.7±0.8 |
| $Cr^{3+}$ | 603.5±4.1 | 88.1±0.6 |
| $Cu^{2+}$ | 745.3±7.5 | 108.8±1.1 |
| $Fe^{2+}$ | 594.6±3.6 | 86.8±0.5 |
| $Fe^{3+}$ | 563.8±3.2 | 82.3±0.5 |
| $Li^{+}$ | 620.6±6.9 | 90.6±1.0 |
| $Mg^{2+}$ | 611.0±8.9 | 89.2±1.3 |
| $Mn^{2+}$ | 663.8±6.6 | 96.9±0.9 |
| $Sn^{2+}$ | 608.3±5.2 | 88.8±0.8 |
| $Sr^{2+}$ | 550.7±8.3 | 80.4±1.2 |
| $Zn^{2+}$ | 626.8±7.6 | 91.5±1.1 |
| EDTA | 667.4±4.9 | 97.4±0.7 |
| SDS | 0 | 0±0 |
| Triton X-100 | 403.5±4.9 | 58.9±0.7 |

a: The enzyme activity was measured using casein (1%, w/v) as substrate at pH 3.0 and 50 °C. All data were mean values ± standard deviations of triplicate tests.

b: The specific activity without metal ions and reagents was defined as 100%.

**Table S4** The expression level and enzyme properties of acid proteases in *K. phaffii* GS115

| Acid proteases | Gene sources | Enzyme activity (U/mL) | Optimal pH | Optimal temperature (°C) | References |
|---|---|---|---|---|---|
| *Ta*proA1 | *Trichoderma asperellum* | 4092 | 3.0 | 50 | This study |
| Apa1 | *Aspergillus niger* | 1500 | 3.0 | 50 | (Wei et al. 2023) |
| PSAPA | *Penicillium* sp. XT7 | 89.3 | 3.0 | 30 | (Guo et al. 2021) |
| PepA | *Aspergillus oryzae* | 50.6 | 4.5 | 50 | (Yue et al. 2019) |
| TAlP | *Talaromyces leycettanus* | 67.8 | 3.0 | 55 | (Guo et al. 2019) |
| RmproA | *Rhizomucor miehei* | 3480.3 | 5.5 | 55 | (Sun et al. 2018) |
| MCAP | *Mucor circinelloides* | 410 MCU/mL (Rennet activity) | 3.5 | 60 | (Kangwa et al. 2018) |
| rP6218 | *Trichoderma harzianum* | 328.1 | 2.5 | 40 | (Deng et al. 2018) |
| PepA | *Aspergillus repens* MK82 | 1.4 | 2.0 | 60 | (Takenaka et al. 2017) |
| TaAsp | *Trichoderma asperellum* | 18.5 | 4.0 | 40 | (Yang et al. 2013) |
